# Origin of mysterious geothermal gas emissions in the middle of the Western Desert, stable shelf area, Dakhla Oasis, Egypt

**DOI:** 10.1038/s41598-023-43492-1

**Published:** 2023-09-30

**Authors:** Mohamed Abdel Zaher, Mahmoud El-Hadidy, Gad El-Qady, Taha Rabeh, Magdy Atya, Sherif El-hady, Abdel Aziz Tantawy, Ibrahim El-Hemaly, Mohamed Al Deep, Ahmed Awad, Hamada Salama, Mohamed Mostafa Khalifa, Mahmoud Leila

**Affiliations:** 1 National Research Institute of Astronomy and Geophysics (NRIAG), Helwan, Cairo, 11421 Egypt; 2 New Valley Vertebrate Palaeontology Centre, New Valley University, New Valley, Egypt; 3 Geology Department, Faculty of Science, Mansoura University, Mansoura, 35516 Egypt

**Keywords:** Environmental sciences, Environmental chemistry

## Abstract

This work responds to what was reported in various audio-visual media channels and to queries and explanations from individuals and local residents on the causes of gaseous and thermal emissions from the Earth near the vicinity of the village of Al-Hindaw in Dakhla city, New Valley Governorate, Egypt. At the location of the fume exit area, magnetic, seismic, and electromagnetic geophysical investigations were carried out to identify the factor(s) responsible for the event in question. Rock samples were collected and studied geochemically and radiographically to assess their chemical compositions, as well as the quantity of organic chemicals that may have contributed to the burning and temperature increase. In light of the results of the geochemical and geophysical research, it is believed that the self-ignitions are the result of near-surface reactions and oxidation instead of volcanic activity, such as the presence of magma or other comparable phenomena.

## Introduction

**Figure 1:**
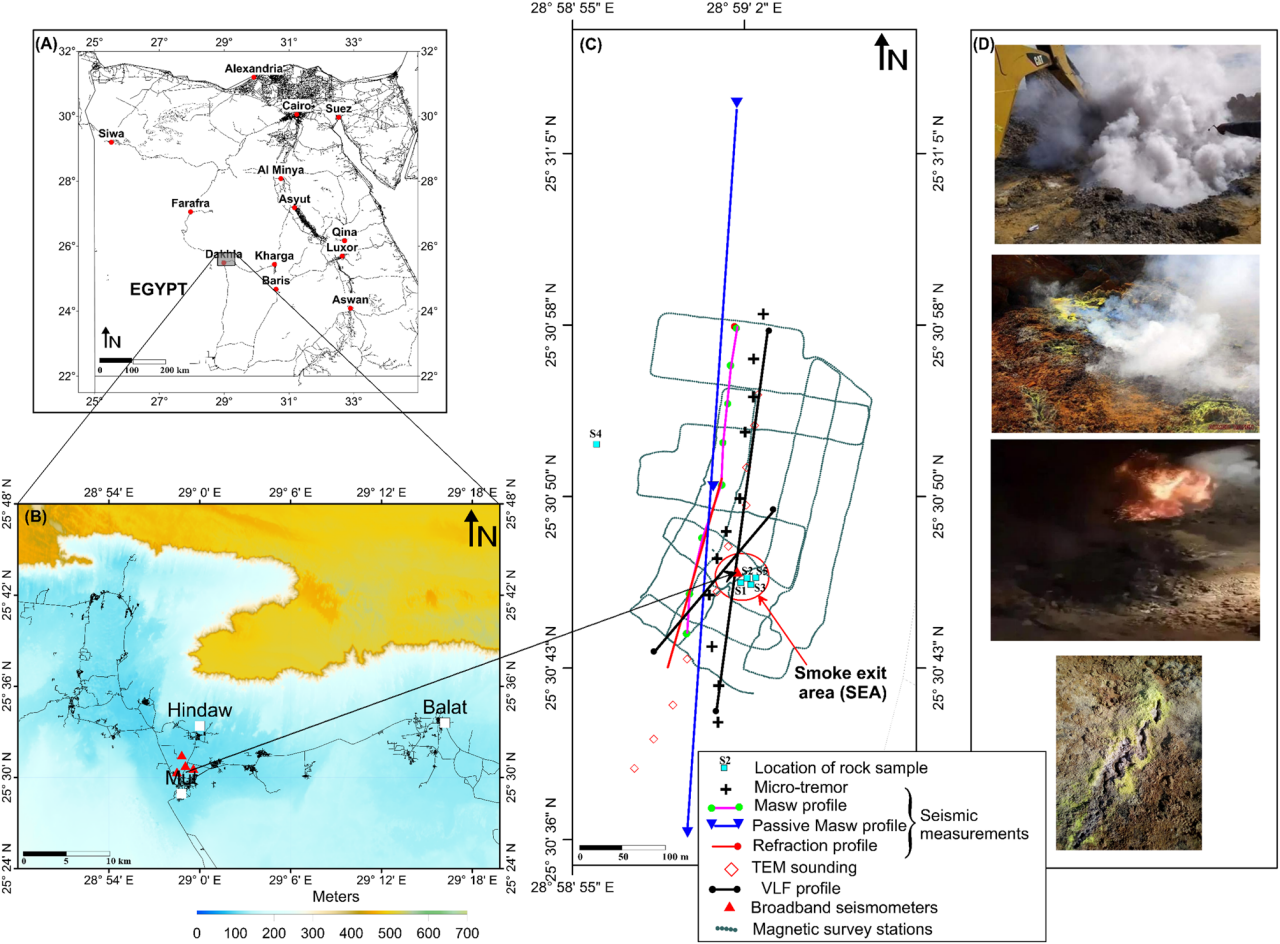
(**A**) A map of Egypt illustrating the position of the Dakhla Oasis in the middle of the Western Desert. (**B**) Topographic map of the Dakhla Oasis relying on a digital surface model (DSM) with an approximate 30-m spatial resolution ^11^. (**C**) The location of geophysical surveys as well as rock samples for geochemical study in/around the smoke exit area (SEA). (**D**) Photographs captured from the locations where smoke and flames are emanating.

The village of Al-Hindaw in Dakhla city (Fig. 1), New Valley Governorate, Egypt, has witnessed a strange phenomenon: the emergence of warm gas emissions (> 100 °C) accompanied by the precipitation of sulfur. It occurs in the area of Al-Hindaw-Mutt Road, approximately 1.5 km from the city of Mut and 2.5 km from the village of Al-Hindaw (Fig. 1). This phenomenon has drawn the attention of various media outlets, which has led to widespread panic among local people. The phenomenon was not confined to this particular locale alone; it was witnessed at more than one location in the general vicinity of the Al-Hindaw region. It commonly arises after excavation work has been done, whether by people or by governments, for the purpose of constructing houses and other infrastructure. Similar hot geothermal emissions have been reported from different places around the world (e.g., Italy, Iceland, Ethiopia, Japan and Mexico). Most of these emissions are associated with hot fluids commonly enriched in CH_4_, CO_2_, N_2_ and H_2_ gases^1–7^. Gas-rich geothermal emissions are often associated with unique geological processes, such as volcanism, fluid‒rock interactions and organic matter maturation ^6,8–10^.

The phosphate mines in Quseir and Safaga experienced self-ignition fires due to their high concentrations of hydrocarbons and pyrite^12^. A significant temperature gradient was observed in the Abu Tarture area (74 m km^-1^), which they attributed to the 80–150 m-deep oxidative heating of pyrite in the phosphate deposit^13^.

Therefore, it was essential to study the phenomenon of self-ignition in the Al-Hindaw area and discover its causes using geochemical and geophysical investigations. A preliminary expedition was conducted as a part of the present research prior to commencing any field surveys. The primary objective of this journey was to acquire comprehensive knowledge about the attributes of the phenomenon being examined, as well as to determine the geophysical methodologies that are the most suitable for the specific region of interest. Consequently, the research methodology was developed based on the geographical situation of the area and the character of the phenomenon being studied. Several geophysical methods were used to conduct field measurements in the study region, including terrestrial magnetic, seismic refraction, multichannel analysis of surface waves (MSW), time-domain electromagnetic (TEM), and very low frequency (VLF) methods. The purpose of these approaches is to establish the kind of rock succession in the area of study, the influence of spontaneous combustion, and the degree to which it extends under the strata, as well as whether there is evidence of subsurface faults, fractures or any seismic activity. Additionally, geochemical studies, organic petrography, and organic geochemical analyses were performed, as well as gas collection and analysis. The results of the geochemical analysis will provide us with information on the chemical and mineral compositions of the rocks, including the percentage of organic compounds present, whether or not radioactive elements are present, and the source of the gases that are being released. The relevance of this study is in understanding the characteristics of the phenomena of natural combustion in order to provide reassurance to the people and facilitate its optimum use. Additionally, it aims to assess the potential risks posed to the local population via an analysis of the released gases.

## Geological and structural settings and seismicity

**Figure 2:**
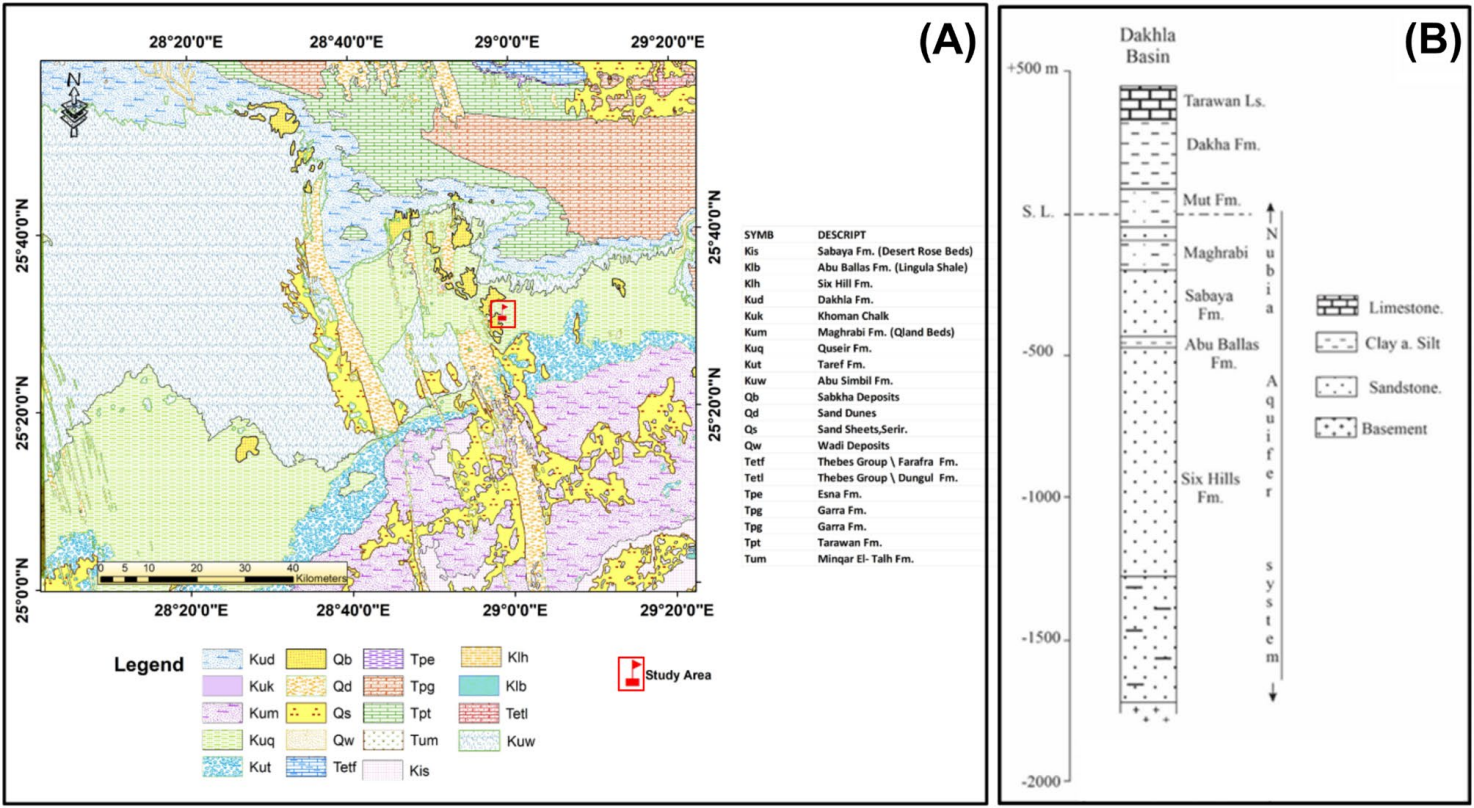
(**A**) Geological map of the Dakhla depression (after E.G.P.C. ^7^). (**B**) Simplified geological section of the Dakhla Basin (after Said ^17^).

The Dakhla Basin, which includes the Dakhla Oasis, is a large sedimentary basin in the middle of the Western Desert. It consists of a very thick and well-exposed Upper Cretaceous–lower Palaeogene succession^14^. The Dakhla Oasis stretches 70 km from west to east and up to 20 km from north to south. Its margins are visible only in the north, where the limestone plateau's massive steep escarpment forms a prominent ridge, while the south is fully open. East of the Dakhla Oasis is a dune-covered plain that extends along the Kharga–Dakhal road^15^. To the north of the Dakhla depression, a high-level limestone plateau reaches a significant distance beyond the oasis^16^. The regional dip of the layers that comprise the Dakhla Oasis is to the north, resulting in the formation of questas along the boundary between the southern Nubian sandstone and the northern Cretaceous shales and Palaeocene chalk. (Fig. 2).

As stated by Said^16^, the area under investigation is a portion of the stable shelf, which mainly consists of the horizontal strata that comprise the Western Desert terrain. Thus, elements of the Dakhla Basin's structure are characteristic of those formed by stable shelf tectonics, which are characterized by gentle tectonic deformations, and the majority of its sedimentary cover is made up of continental as well as epicontinental deposits. Said ruled out tectonic origins for the Western Desert depressions and thought that the Dakhla depression was located in plateaus that did not experience considerable tensional strains during ascent^18^. According to Klitzsch et al.^19^, the average thickness of the Nubian Group on the southern edge of the Dakhla Basin lies between 1000 and 2000 m, and the sequence is Jurassic–Cretaceous in age. The layers are unconformably deposited on the Precambrian basement or, more locally, on Palaeozoic rocks in the west.

The study area's surface geology reveals many layers of the variegated shale that emerge on the surface and are covered in some places by layers of the Dakhla shale and in others by layers of the Tarawan Formation. Additionally, the region is defined by the existence of many fractures and warps in a variety of directions, the majority of which run northeast‒southwest and perpendicular to it, i.e., northwest‒southeast, which is consistent with similar patterns seen in other regions within the Western Desert^20–22^.

Before 1987, neither historical nor instrumental seismicity was recorded near the southwestern corner of the Western Desert. An earthquake with magnitude mb = 5.3 occurred on the El-Gilf El-Kebier Plateau on December 9, 1987. El-Baz et al.^23^ investigated the geology of the region and found that it is influenced by two faults: the Gilf fault, which strikes N‒S for 150 km within Egypt and continues southwards into Sudan, and the Kermal fault, which restricts the plateau's northwestern edge and strikes NW‒SE. The Kemal fault crosses the northern end of the Gilf fault.

## Field surveys and data acquisition

### Geophysical surveys

In the area under investigation, field measurements were conducted using different geophysical tools, including land magnetic, seismic, TEM and VLF techniques. The magnetic total-field data were collected with a GSM-19 (Overhauser) and a base station for diurnal corrections. Continued measurements were acquired to cover the entire area around the smoke exit area (SEA). A comprehensive terrestrial magnetic survey was conducted in the Al-Hindaw region. Two Overhauser magnetometers were utilized: one was used as a stationary base station in various homogeneous locations, and the other was used for making observations approximately 0.5 to 1 m apart, depending on the walking speed. After correcting the data for diurnal and annual fluctuations, a map of the total magnetic field was created.

The TEM measurements were taken at ten points along the N‒S profile that traverses the SEA. The distance between adjacent stations was between 100 and 200 m. The TEM data were acquired using a SIROTEM MK3 conductivity meter with a single loop design and a 50 m loop side length to reach a depth of approximately 150 m under the Earth's surface. The data were captured in composite mode using up to 30 discrete time intervals (time gates).

A total of 104 measurement points were observed along two lines utilizing the T-VLF device with an interval of 5 m between each station, as shown in Fig. 1. We used two transmitters with frequencies of 17,900 and 24,000 Hz. These transmitters give well-observed radio signals and portrayed a narrow azimuthal distribution. Typically, the tilt angle and ellipticity of the vertical magnetic polarization ellipse were deduced from the estimation of two orthogonal magnetic field components.

Additionally, three seismic shallow refraction profiles were acquired in a north‒south direction, each of which had a length of 151 m with a total length of 453 m and passed by the SEA. Each line was shot many times in order to obtain good records, which allowed recording the largest possible time of arrival of seismic waves, as well as obtaining a high-resolution model of the underlying structures in the research area. Six surface wave profiles were also recorded to obtain surface waves. The acquisition of surface waves was similar to the acquisition of traditional shallow seismic refraction profiles where the geophones are aligned in a straight line with equal geophone spacing. In the current study, a 48-channel seismograph and 4.5 Hz geophones were used. The geophone spacing that was used was 1 m. The roll-along acquisition technique was implemented, where only 24 channels were active during each shot. The shot was placed − 4 m from the first geophone. The shot process was shifted 4 m and repeated eight times. Figure 1 illustrates the locations of these profiles. In addition, two profiles of passive MASW were recorded using 48 geophones with 5-m intervals and a total length of 235 m to reach deeper parts. Finally, Microtremor measurements were acquired in the study area for 12 sites in a north‒south direction with distances between 100 and 50 m. The data were recorded using Trillium 40 and Trellium 120 s seismometers (Nanometrics Inc.). The length of recording was approximately 2 h at each site.

In addition, continuous temperature logs were recorded using a GeoVista PT100 Sensor^24^ in a downwards direction in two wells that are located close to the SEA. The probe that was used was designed to produce precise temperature readings in a borehole environment with a resolution of 0.01 °C.

### Geochemical surveys

#### Rock sampling

Five different rock samples were collected from the research region (Fig. 1); four samples were taken from the SEA, and one sample was taken from an area that was 110 m to the northwest of the SEA. This location was once home to the same phenomena, but it no longer exists. During the process of collecting samples, extreme care was used to guarantee that they were not damaged by corrosion or by being burned. The samples were analysed on LECO SC 632 equipment at the Egyptian Petroleum Research Institute (EPRI), Cairo, Egypt, to measure their total organic carbon (TOC) and total sulfur (TS) concentrations following the procedure described by Prinz et al.^25^. The analysis consisted of burning nearly 100 mg of the powdered sample in the presence of oxygen, where the combustion temperature was increased gradually from 100 °C to more than 1000 °C. Moreover, X-ray fluorescence (XRF) measurements were conducted in EPRI on five powdered samples to obtain their elemental composition (major elements) using an Axios Sequential PANalytical 2005 WD_XRF Spectrometer.

Organic petrography was performed on two samples with the highest TOC contents to qualitatively estimate the kerogen composition following the procedure described by Makled et al.^26^. For kerogen separation, 10 g of powdered sample was treated with HF and HCl acids, and the residues were screened via a 200-m brass sieve and then a 10-μm nylon sieve. Moreover, the organic kerogen was separated from the residue using a solution of zinc chloride and then mounted on glass slides for microscopic analysis. Additionally, pyrolysis gas chromatography (Py-GC) analysis was conducted on the whole rock powder to examine the chemical composition of the organic kerogen. The obtained gas chromatograms often illustrate the major components within the organic kerogen and hence fingerprint the kerogen type, origin, and composition.

#### Gas sampling

Seven locations in the research region were sampled for gas emissions using pre-emptied 20 ml glass bottles and a silicone funnel linked to a silicon hosepipe. In the ISOLAB BV research laboratories in the Netherlands, gas chromatographic (GC) analyses were carried out to evaluate the molecular structure of the collected gases. The C_1_, O_4_, A_r_ and N_2_ contents and their gas emissions were determined via a heated injection valve and a thermal conductivity detector (TCD) and an Agilent 6890 N/7890A/7890B GC (Agilent Technologies, Santa Clara, CA, USA) outfitted with a 12 m and 0.32 mm molsieve column (Agilent). The C_2_^+^, CO_2_, and CO concentrations were evaluated utilizing an Agilent 6890 N/7890A/7890B GC outfitted with a heated injection valve, TCD, and FID, as well as a 50 m and 0.32 mm Porabond-Q column (Agilent). The readings were routinely calibrated using synthetic gas mixes with known compositions throughout the day. The O_2_, N_2_, and Ar gas readings were calibrated with atmospheric air. The detection thresholds for hydrocarbons are 1 ppm, whereas those for CO_2_, O_2_, and N are 100 ppm. The findings of compositional analyses of the examined Dakhla gas leaks were adjusted for air contamination by employing the (N_2_/O_2_) air and (Ar/O_2_) air values of 3.70 and 0.048, respectively, and the observed gases were given in mol%.

The carbon δ^13^C composition was measured for CO_2_, as well as the C_1_–C_3_ hydrocarbon phases at the four sites that have the highest hydrocarbon gas contents, whereas the deuterium δ_D_ of methane was measured for only one sample. In addition, nitrogen δ^15^N isotopic values were also measured for the same sites. At least three measurements were performed for each sample, and the average result was then reported. For δ^13^C isotope values in hydrocarbon phases, an Agilent 6890 N GC was connected to a Finnigan Delta S IRMS through a Finnigan GC-C II interface. For the determination of δ^13^C values in CO_2_, an Agilent 7890A GC interfaced to a MAT 253 IRMS through a GC-Isolink or Finnigan GC-C III interface was used. The values for carbon isotopes were provided with a maximum experimental error of (1σ) of ± 0.2‰ compared to ‰VPDB. The deuterium values for methane were acquired using an Agilent 7890A GC coupled to a MAT 253 IRMS, and the δ_D_ values were given relative to ‰VSMOW, with an experimental error (1σ) of no more than ± 5%. Using an Agilent 7890A GC (interfaced to a Finnigan MAT 252 IRMS via a connected Finnigan GC-C II), the δ^15^N values for nitrogen in the examined gases were measured. The δ^15^N readings were routinely calibrated with nitrogen from ambient air, and the results were published with an experimental error (1σ) of ± 0.2‰ relative to atmospheric N_2_.

## Methodology and results

### Geophysical data

#### Geomagnetic data

**Figure 3:**
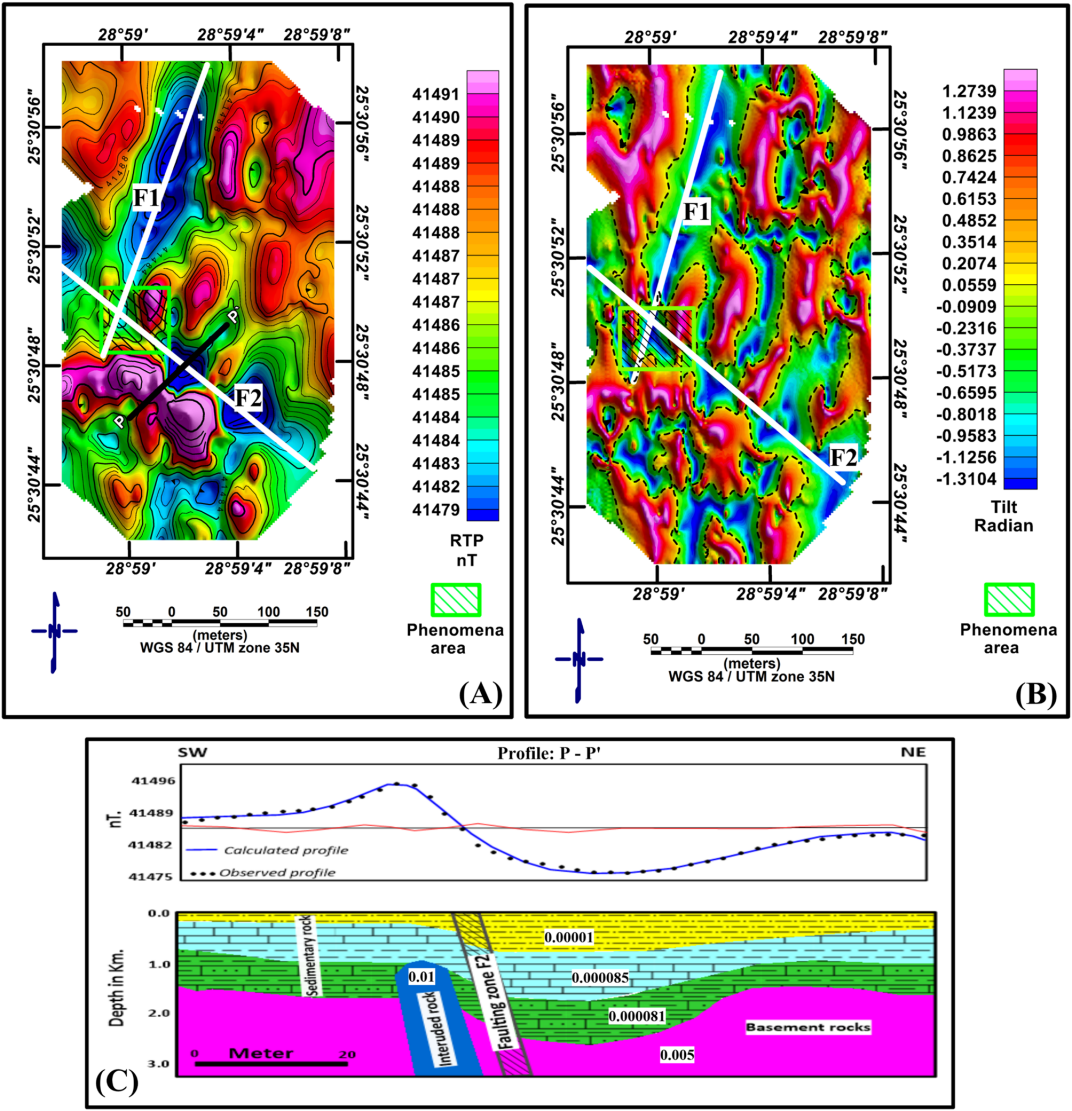
(**A**) RTP land magnetic map of the AL-Hindaw area. (**B**) Tilt derivative RTP magnetic anomaly map, where the yellow square delineates the SEA area. (**C**) 2D magnetic modelling along profile P-P´. The numbers in the modelled section refer to the magnetic susceptibility values in SI units.

On the surveyed total magnetic intensity (TMI) map, the reduction to the pole (RTP) approach was employed to rectify the shift between sources and magnetic anomalies induced by the nonverticality of the normal field and magnetization. The inclination of the normal field was adjusted to 42.4 north, while the declination was set to 2.5 east^27^. The RTP terrestrial magnetic map of the AL-Hindaw region (Fig. 3A) shows that the majority of the anomalies are oriented NE‒SW, NW‒SE, and E‒W. Positive magnetic anomalies are also visible in the eastern and northwest regions of the map, while negative anomalies are found in the centre. The tilt derivative magnetic anomaly map (Fig. 3B) clearly shows that the region is influenced by two fault systems, F1 and F2, which run NE and NW, respectively, and meet at the SEA.

Based on the theories of Talwani et al.^28^, a 2-D modelling approach was applied to selected profiles on the RTP map utilizing GMSYS-3D modelling software^29^. The 2D inversion model enables us to handle both layered structures and single objects, such as igneous intrusions. The approach is based on the correlation between the observed magnetic curve from the RTP magnetic map and the predicted curve. The magnetic susceptibility was the variable factor in the procedure that produced more or less full matching between the observed and computed curves. Within layers, magnetic susceptibility may vary both vertically and horizontally. Each causal body has a distinctive potential field that can be built by adjusting specific factors, including its extent, magnetic susceptibility, inclination, declination and x–z positions. On the basis of drilled wells and previously published depth estimations, we suggest that the sedimentary rock above the basement comprises three distinctive rock units. In the bottom layer, the two limestone rocks prevail, but in the upper layer, the shale deposit rocks predominate. By adjusting the magnetic susceptibility coefficients of the subsurface rocks and their accompanying intrusion as well as the depths of the basement layer, it is possible to match the estimated and observed magnetic curves, whereas the computed model's root mean square (RMS) value is 0.55. Figure 3C depicts the resulting model, in which it can be shown that fault structure F2 has been downthrown approximately 400 m.

#### Seismic data

##### Shallow seismic refraction

**Figure 4:**
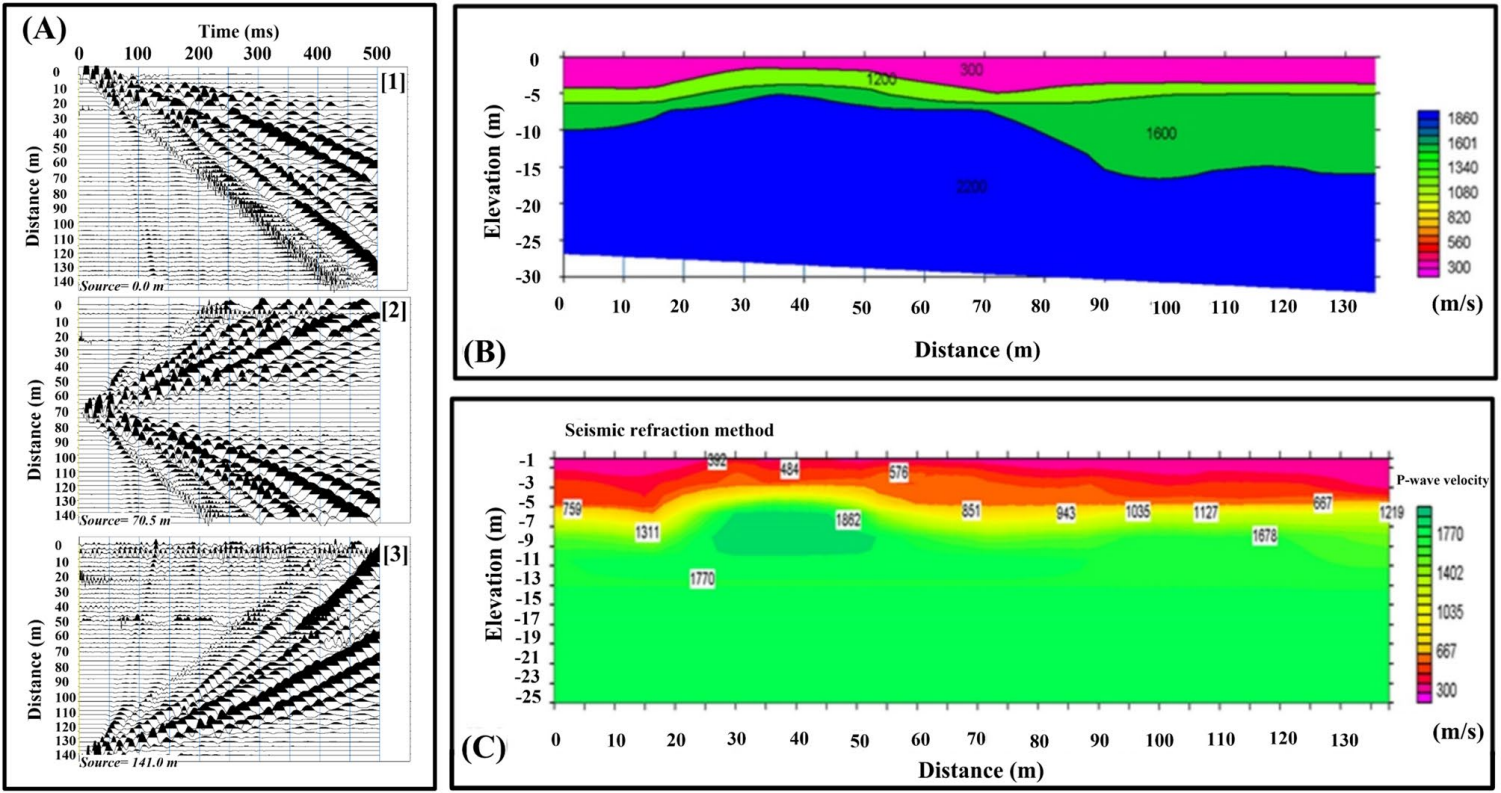
(**A**) Examples of the obtained seismic records, (**A-1**) Normal shot, (**A-2**) Mid shot and (**A-3**) reverse shot; (**B**) Stratified model determined by time–term inversion (initial model); (**C**) the obtained seismic tomographic inverted model of the emission area.

In this study, three shallow seismic refraction profiles were measured in the emission area. The data were processed, and a 2-D subsurface tomographic model of the study area was obtained from the arrival time inversion. The processing was performed first using the initial model obtained by the time–term inversion technique, which was the input for the tomographic inversion using SeisImager software. All interpretation theory and practice methods are summarized in Geometrics and OYO SeisImager/2D^TM^manual, 2009 Ver. 3.3^30^. Figure 4 provides examples of a stratified model determined by time–term inversion (initial model) and tomography inverted model (second step). The third profile barely passed over the emission area location. The obtained model shows that the study area comprises four layers with P-wave velocities between 300 m/sec and 1890 m/sec. The obtained model shows that the surface layer is removed at the emission site, and two faults might bind the emission site.

##### Multichannel analysis of surface waves (MASW)

The MASW method^31–34^ is a widely used approach for imaging subsurface structures^35^. The analysis of the acquired waveforms depends on measuring the dispersion of the recorded Rayleigh waves. Six MASW profiles were measured in the SEA, as shown in Fig. 1.

**Figure 5:**
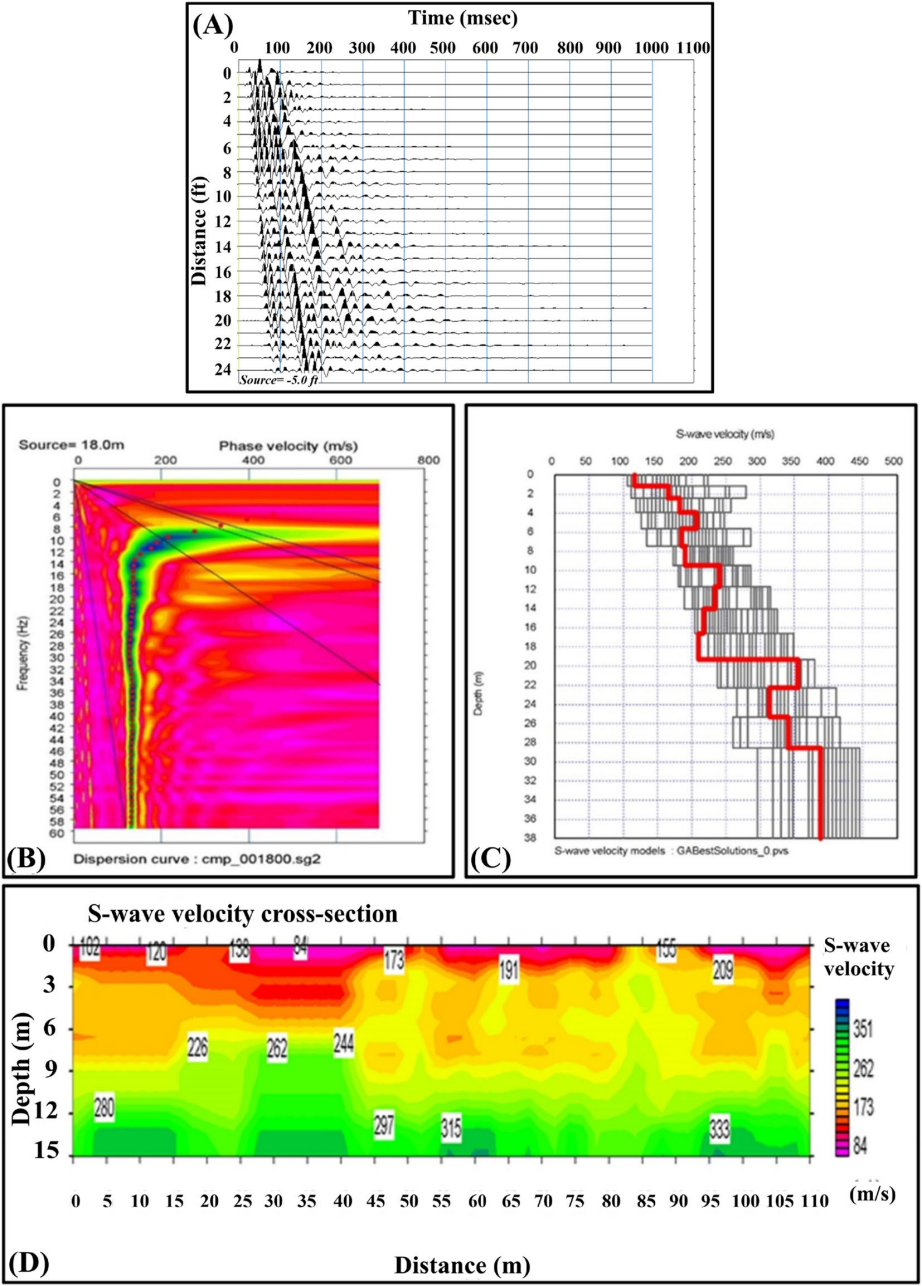
(**A**) Example of the obtained seismogram; (**B**) Example of the obtained dispersion curves; (**C**) example of the obtained 1-D model; (**D**) the obtained 2-D shear wave velocity model.

The processing of the acquired profiles was performed using a genetic algorithm. This algorithm has several advantages over traditional processing procedures^36^. Figure 5B shows an example of the obtained curves. A 1-D subsurface model was obtained for each shot (Fig. 5C), and then a 2-D subsurface model was obtained. Figure 5D shows a merging of three MASW profiles passing beside the emission site. The processed shear wave velocity model shows that the area comprises four strata with shear wave velocities ranging from 100 to 333 m/sec. The disappearance of the surface layer at the emission location and the faulting impacting the region are both visible in the 2-D profile.

##### Passive MASW

**Figure 6:**
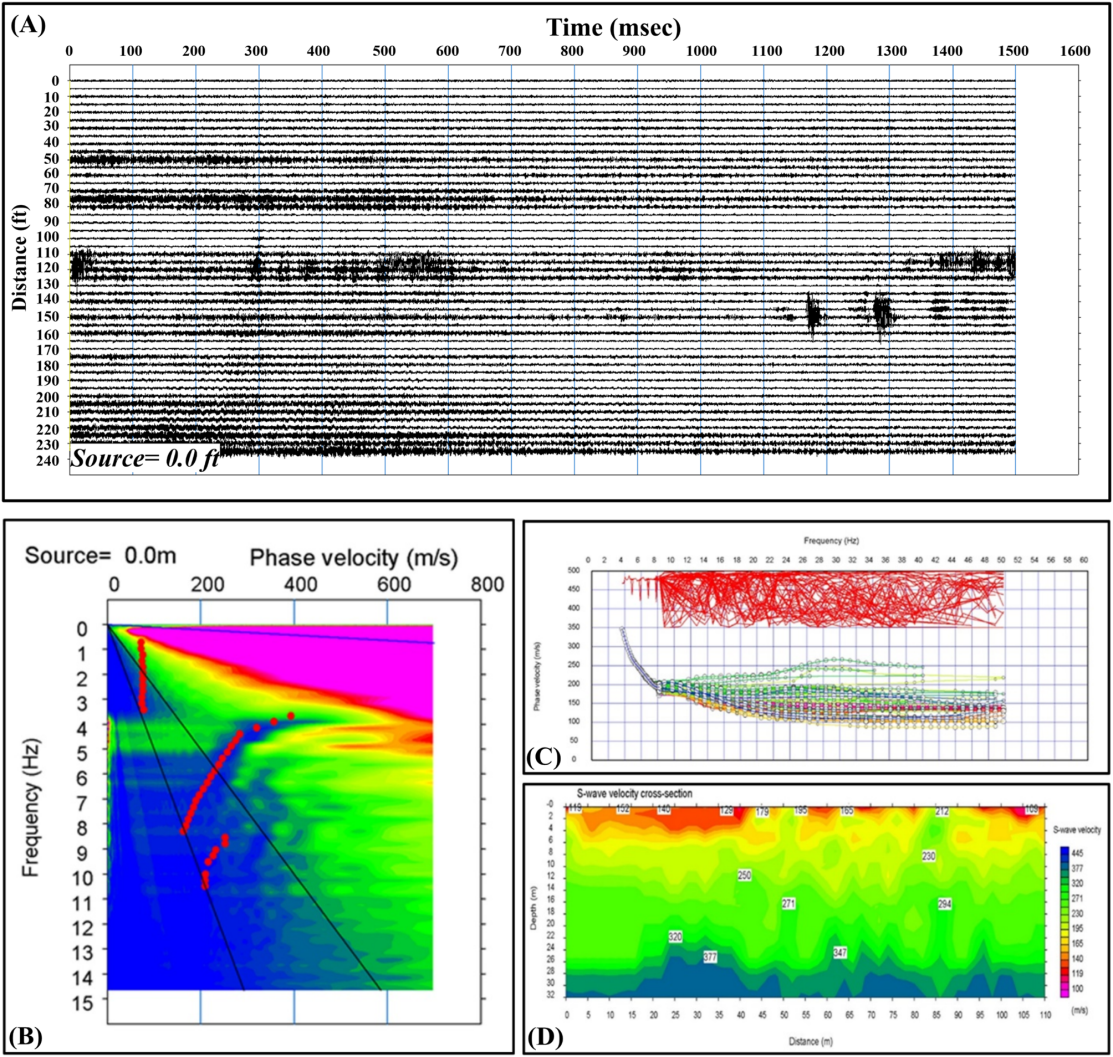
(**A**) Example of the obtained passive seismogram; (**B**) Illustration of a dispersion curve produced from passive MASW measurements; (**C**) example of the merged dispersion curve generated from active and passive MASW; and (**D**) the subsurface model derived by integrating the active and passive MASW profiles.

This method has been used many times worldwide by many scholars ^32,37–39^. Two passive MASW profiles are acquired at the site. An example of the obtained dispersion curves is shown in Fig. 6B. For deeper subsurface imaging, the acquired dispersion curves are combined with those from the MASW. The merged curves are illustrated in Fig. 6B. Figure 6D demonstrates the shear wave subsurface model of the study site utilizing both the dispersion curves obtained from MASW and passive MASW. The last figure indicates much heterogeneity appearing close to the surface compared to the deeper part of the merged section. Figure 4 shows that the P-wave velocities in the study area are 300 m/sec, 1200 m/sec, 1600 m/sec and 2200 m/sec for the geoseimic layers 1, 2, 3, and 4. On the other hand, Fig. 5 shows a shear wave velocity profile, where the shear wave velocity is 150 m/sec in the first layer, 200 m/sec in the second layer and 350 in the third layer. The obtained Vp/Vs in the study area ranges between 2.0 and 4.0. These high values of the Vp/Vs can be attributed to the gas occurrence and associated high temperature.

##### Microtremor analysis

**Figure 7:**
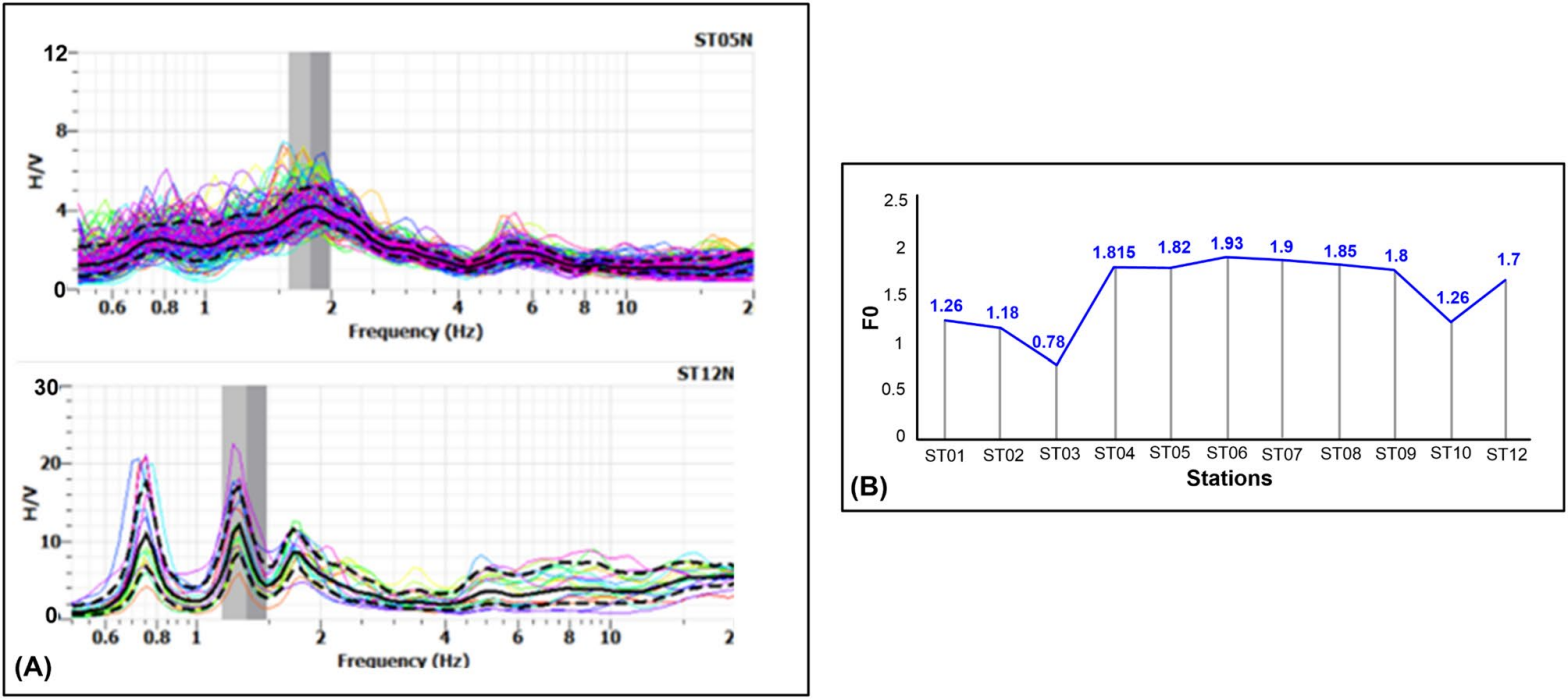
(**A**) The examination of microtremor data from sites 5 and 12. (**B**) The spatial distribution of the obtained F_0_ values.

The seismic noise measurements were acquired close to the emissions at 12 sites with interstation distances of 10 and 20 m, as shown in Fig. 1. The aim of these measurements was mapping the variation in the dominant frequency (F_0_) using the HVSR technique ^40^, where the change in the value of F_0_ reflects the difference in the subsurface structures ^41–43^. The data were analysed using Geopsy ^44^. Figure 7A shows the analysis applied to the acquired data.

The analysed data show that the F_0_ values within the study area range between 0.78 and 1.93 Hz, with a sharp change in F_0_ values between stations ST03 and ST04 and ST09 and ST10 (Fig. 7B). Usually, these differences may be attributed to faulting.

##### Local seismic network

A temporary network of 4 broadband seismometers was installed with the other Egyptian National Seismological Network (ENSN) stations near the study area (Fig. 1). Establishing such networks aims to monitor microearthquakes, which may be associated with this phenomenon. The network was operated for 2 weeks during and after the emission. During the installation of these stations, no seismic activity was recorded, which could be related to the phenomenon being investigated.

#### TEM data

**Figure 8:**
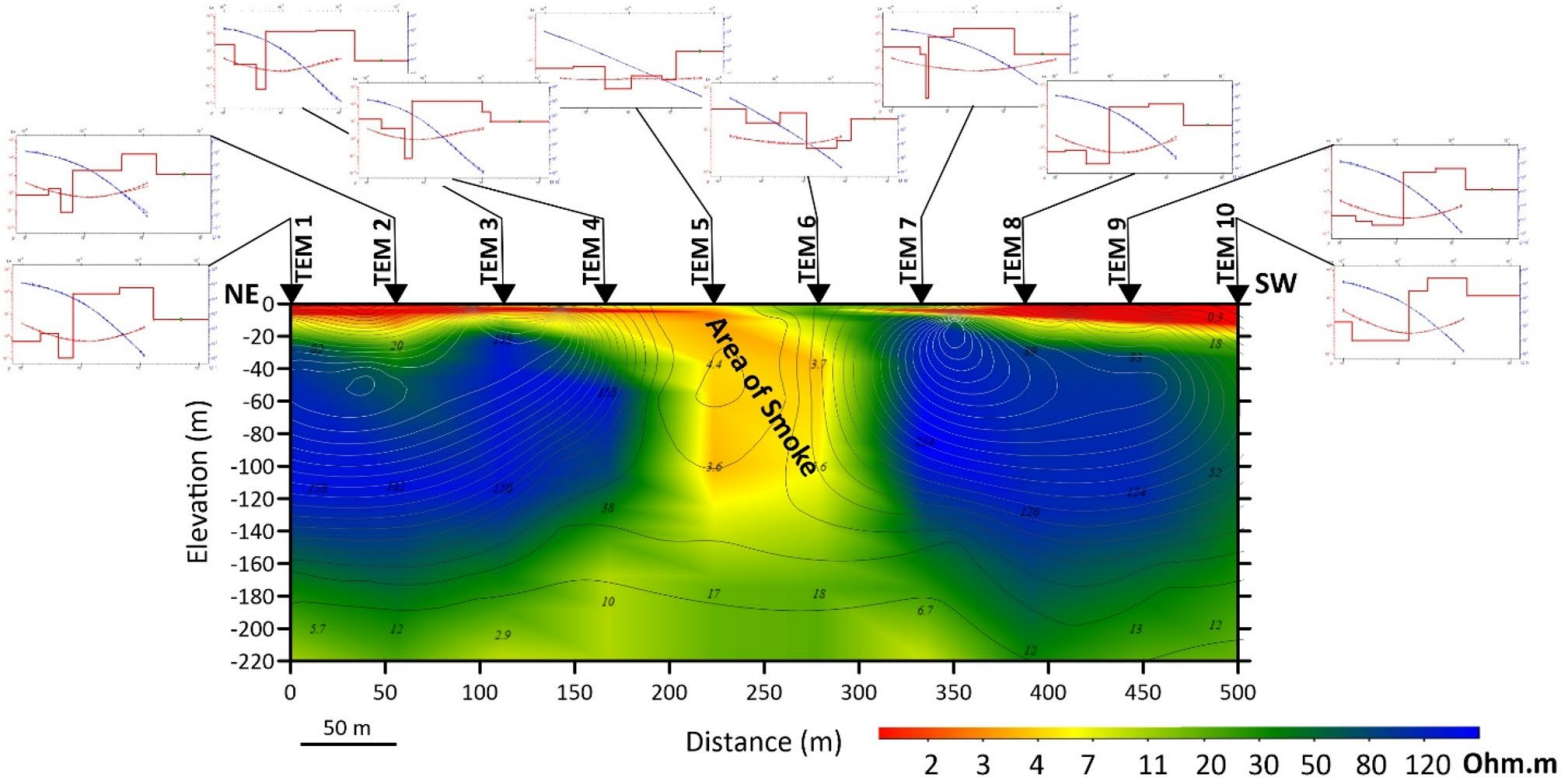
Geoelectrical cross-section of the TEM data along the profile passing through the smoke exit area (SEA). The locations of the TEM soundings are shown in Fig. 1. The above graphs refer to the results of 1-D modelling of TEM data.

Using the ZONDTEM1D program^45^, analyses and modelling of TEM data were performed. A range of criteria, including the number of layers and their thicknesses, were evaluated to determine the best possible starting model for TEM data processing. The robust model is the model with the least amount of error, the best fit, and the greatest compatibility with the given geological data. The procedure was carried out in a refined manner until a composite model satisfying the TEM data was produced. Each model characterizes the geoelectrical properties of the underlying layer at its corresponding location. The obtained models have been utilized for generating a north‒south geoelectrical cross-section, as shown in Fig. 1. The geoelectrical section constructed in the study region is illustrated in Fig. 8.

The geo-electrical cross-section shows that the area where smoke and high temperatures appear is characterized by a decrease in electrical resistivity compared to the rest of the areas, which indicates the effect of high temperatures on electrical conductivity, leading to its warming up and thus a reduction in electrical resistance.

#### VLF data

**Figure 9:**
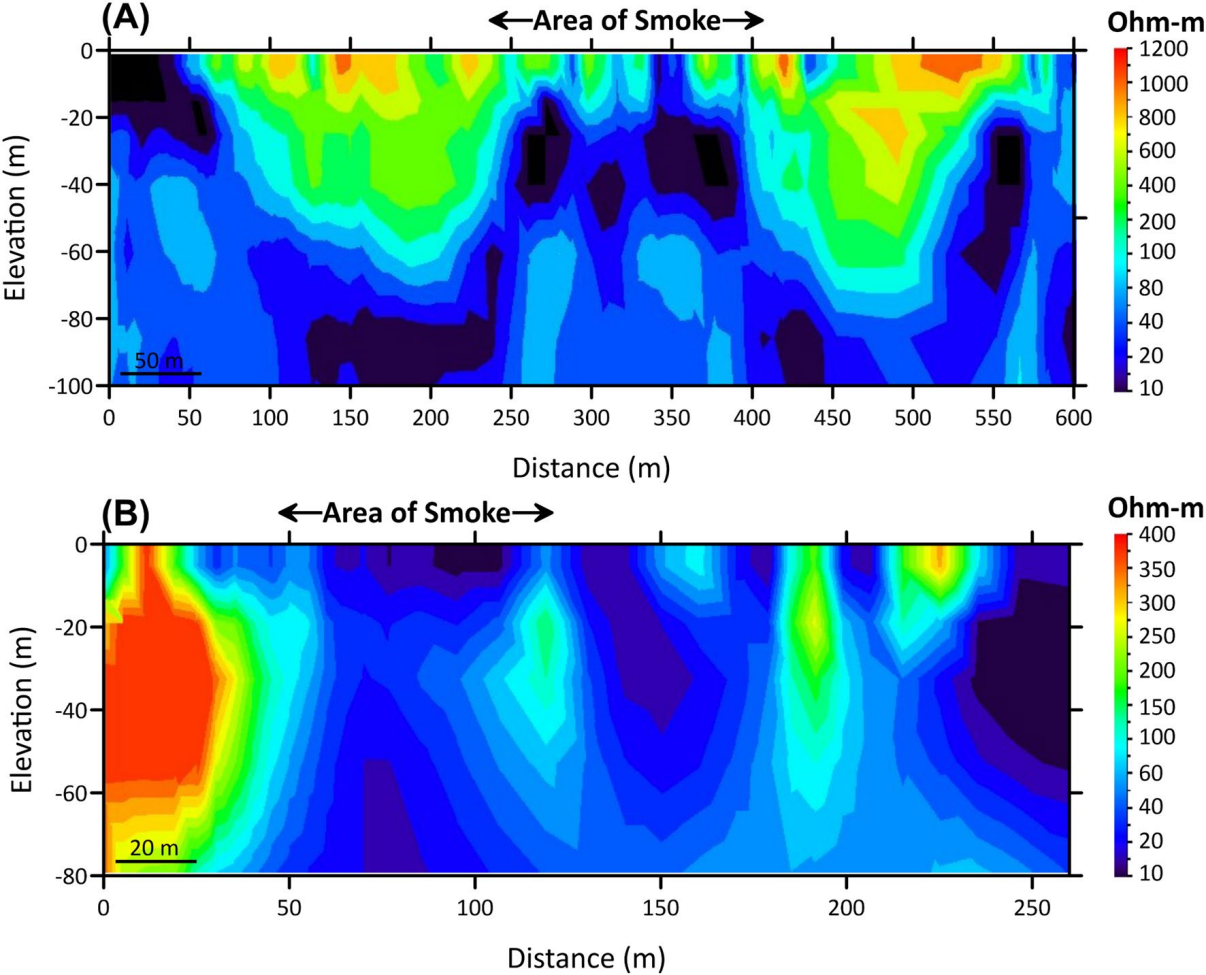
Modelled resistivity cross-sections of the VLF data along (**A**) profile 1 and (**B**) profile 2, both of which pass through the SEA.

The processing of the VLF data aims to calculate the subsurface current density distribution. The data are processed using the VLF2DMF program^46^ to calculate the K-H filter, and then inverse modelling is applied to estimate the subsurface resistivity distribution. As illustrated in Fig. 1, the first profile extends from north to south, while the second profile extends from northeast to southwest. Both profiles intersect the SEA. The K–H filter^47^ in both profiles demonstrates a significant amount of variance in the real component amplitude in excess of the SEA. In addition, the resistivity model demonstrates a very low resistivity anomaly beneath the smoke area. The VLF data lend credence to the conclusion drawn from the TEM data, which is that an increase in the rock temperature results in an increase in the rock electrical conductivity (Fig. 9).

#### Temperature logs

**Figure 10:**
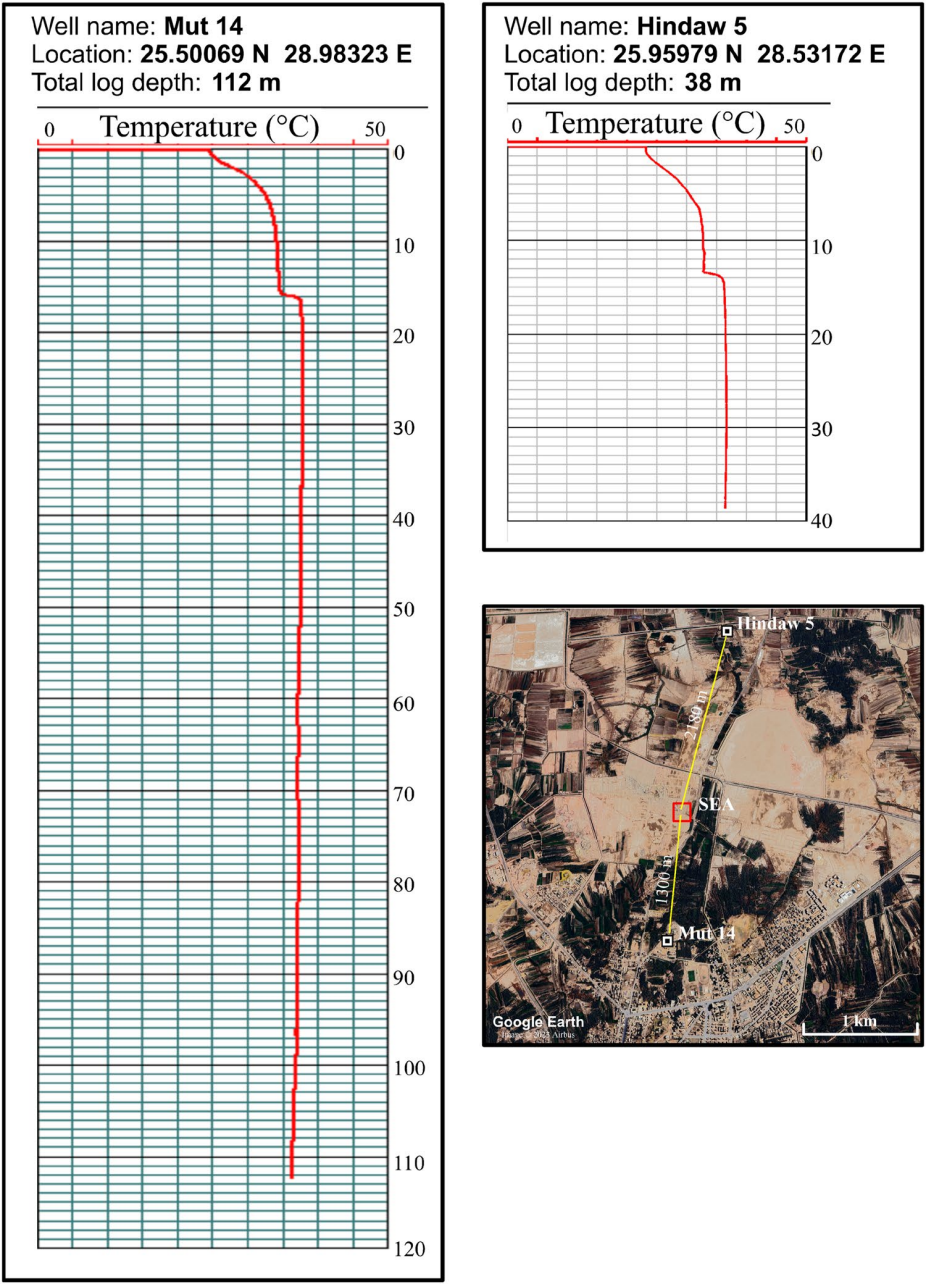
Temperature recording using the GeoVista well logging instrument in two water wells near the SEA. The Google Earth map shows the location of these wells from the area of smoke.

Temperature logs are able to provide information on the change in temperature as the probe traverses deeper, and as a result, the temperature gradient in the measured well can be determined. The log recording in well Mut 14, located 1300 m south of the SEA, attained a maximum depth of 112 m. At this depth, a temperature reading of 37 °C was recorded. On the other hand, the log recording in well Hindaw 5, which is situated 2150 m north of the SEA, reached a maximum depth of 38 m. A temperature measurement of 36 °C was made at this depth (Fig. 10). The trend of the temperature curves in both wells demonstrates that there is no discernible change in temperature with depth, which implies a low temperature gradient (approximately 0.025 °C/m in well Hindaw 5 and 0.022 °C/m in Mut 14).

### Geochemical data

#### Elemental analysis

Data from elemental geochemical analyses of the five obtained rock samples are shown in Table 1. The ranges of TOC and TS values in the samples are between 0.05 and 4.29 wt% and 0.09 and 12.6 wt%, respectively. The shales are enriched in Al_2_O_3_ (7.85–19.1 wt%), SiO_2_ (19–61 wt%), and Fe_2_O_3_ (5.75–26.5%), whereas all other oxides have reported values less than 5%. Notably, the shales are highly depleted in Na_2_O, with values in the range of 0.08–0.46 wt%. Similarly, the shales contain traces of MnO (0.05–0.24 wt%), whereas the concentrations of CaO and MgO are variable and range from 0.48 to 3.11 wt% and 1.25 to 3.75%, respectively. Furthermore, the contents of P_2_O_5_ vary in a wide range from 0.12–1.84 wt%.

**Table 1:** Elemental composition of the studied Dakhla shale samples.

Sample	TOC	Na_2_O	MgO	Al_2_O_3_	TiO_2_	K_2_O	Fe_2_O_3_	CaO	MnO	TS	SiO_2_	P_2_O_5_
S_1_	0.06	0.193	1.27	14.9	0.77	1.82	12.8	1.22	0.059	0.3	61	1.17
S_2_	0.27	0.08	1.76	19.1	1.07	2.25	5.75	0.481	0.05	1.29	58.3	0.28
S_3_	0.05	0.09	3.75	15.4	1.09	3.38	9.74	3.11	0.16	0.09	51.9	1.84
S_4_	0.71	0.12	1.3	17.4	1.69	2.79	11.1	0.55	0.04	0.62	51.1	0.12
S_5_	4.29	0.46	1.25	7.85	0.44	0.99	26.9	1.43	0.24	12.6	19	0.32

#### Organic petrography

**Figure 11:**
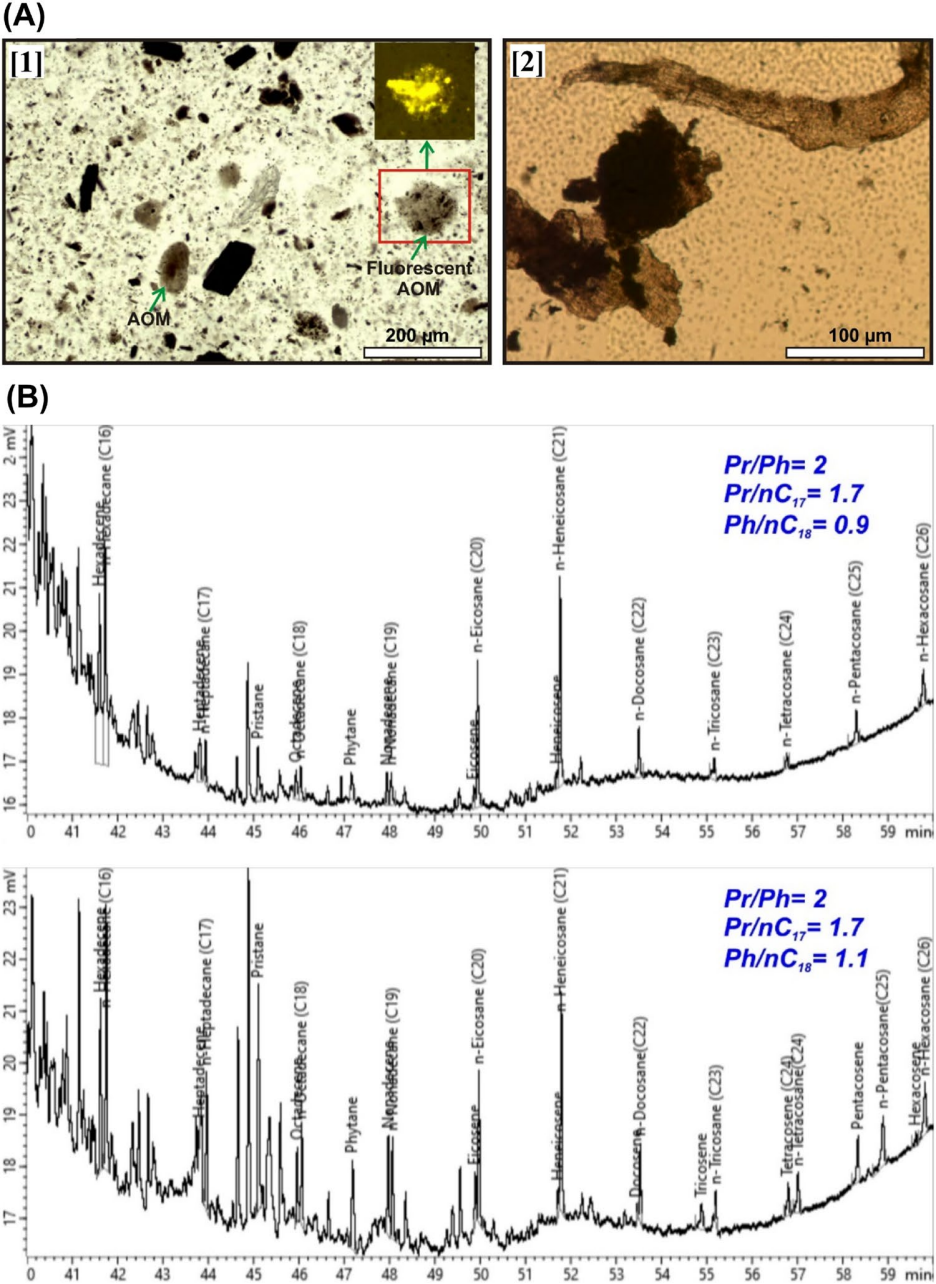
(**A**) Thin section microphotographs showing the composition of the Dakhla shale kerogen with abundant fluorescent amorphous organic matter [1] and cuticles [2]. (**B**) Gas chromatograms illustrating the n-alkane distribution in the analysed bitumen extracts.

The analysed samples (S_4_, S_5_) with the highest TOC values are less degraded and contain well-preserved organic matter. Fine-grained fluorescent and nonfluorescent diffuse brown and opaque amorphous organic materials (Fig. 11A[1]) and fluorescent cuticles (Fig. 11A[2]) are the most abundant components. The terrigenous organic matter is represented by lath-shaped translucent brown phytoclasts. Palynomorphs and vitrinite fragments are absent. The preserved organic matter points to abundant type II and blended type II/III kerogen.

#### Molecular composition of bitumen extracts

Extracted bitumen from the S_4_ and S_5_ samples displays relative enrichment in n-alkanes in the range of C_1_–nC_15_ (> 90%). Alkanes (~ 60%) and aromatics (~ 20%) are the most abundant components, whereas isoparaffins (~ 6%) and naphthalenes (~ 5%) are comparatively less abundant (Table 2). The samples have similar isoprenoid pristine/phytane Pr/Ph and isoprenoid/n-alkane (Pr/nC_17_, Ph/nC_18_) values (Fig. 11B).

**Table 2:** Geochemical characteristics of the bitumen extracted from the Dakhla organic-rich shales.

Sample	C_1_–C_5_%	nC_6_–C_14_%	nC_15+_%	Pr/Ph	Pr/nC17	Ph/nC18
S_4_	54	38	8	2	1.7	0.9
S_5_	58	36	15	2	1.7	1.1

#### Gas chemistry

The primary phases shown in the examined gas manifestations are gaseous N_2_, CO_2_, Ar, CO, and CH_4_ (Table 3). N_2_ is the most abundant gas phase, varying in a narrow range from 86.32 to 88.53 vol%. CO_2_ is the second most abundant gas phase, with a narrow range of concentrations from 10.27 to 11.23 vol%. Argon is present in significant concentrations from 1 to 2.28%. Traces of CO are recorded from 0.09 to 0.15 vol%.

**Table 3:** An analysis of the chemical and isotopic contents of gas seeps from seven locations throughout the research region.

Site	C_1_	C_2_	C_3_	C_6+_	N_2_	CO_2_	CO	Ar	δ^13^C_CO2_	δ^13^C_C1_	δD_C1_	δ^13^C_C2_	δ^13^C_C3_	δ^15^N
1	0.03	0.00	0.00	0.03	87.83	10.85	0.15	1.10	–	–	–	–	–	
2	0.03	0.00	0.00	0.01	88.53	10.27	0.09	1.07	− 25.5	− 29.6	–	− 28.9	–	− 0.1
3	0.06	0.00	0.00	0.02	87.24	11.45	0.14	1.09	–	–	–	–	–	
4	0.06	0.00	0.00	0.01	87.46	11.23	0.16	1.08	− 25.4	− 24.8	-	-25.5	− 27.2	− 0.2
5	0.06	0.00	0.00	0.02	87.36	11.37	0.14	1.05	–	–	–	–	–	
6	0.06	0.00	0.00	0.01	86.32	11.18	0.14	2.28	− 25.4	− 24.2	–	− 25.3	− 26.5	− 0.4
7	0.41	0.06	0.05	0.14	87.25	11.06	0.00	1.00	− 13.9	− 24.1	− 160	–	–	− 0.5

**Figure 12:**
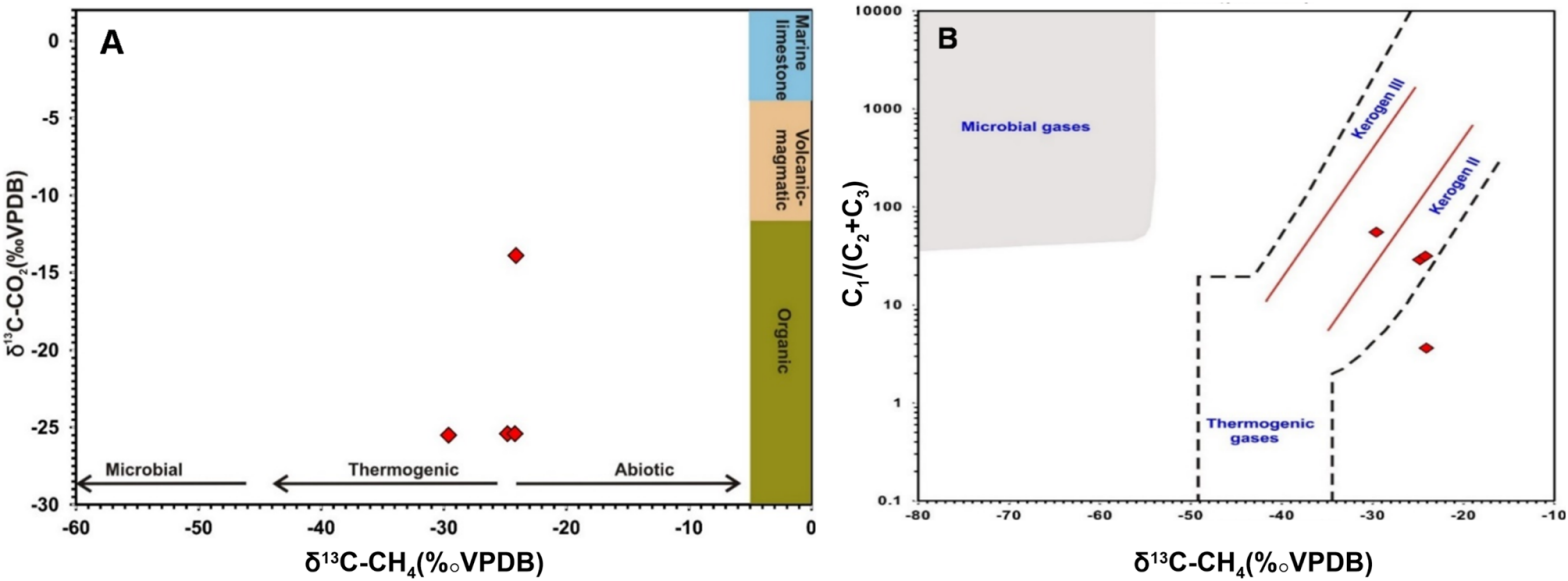
(**A**) δ^13^C_CH4_ versus δ^13^C_CO2_ cross-plot illustrating the organic origins of both CO_2_ and CH_4_ gases; (**B**) δ^13^C_CH4_ versus the molecular C_1_/(C_2_ + C_3_) ratio showing that the vast majority of the gas samples are grouped together in the organic thermogenic zone of Bernard et al., 1978^48^.

Hydrocarbon gases are mainly represented by methane (0.025–0.41 vol%), ethane (0.001–0.064 vol%), and propane, with a maximum recorded value of 0.04 vol%, and the C6 + components vary from 0.01 to 0.14 vol%. The δ^13^C_CO2_ values vary in a wide range between − 25.5 and − 13.9‰ (Fig. 12). The δ^13^C rates of methane, ethane and propane are quite identical and heavier than -30 ‰ and display offsets of less than 3‰. The δD of methane was measured at one site, revealing a value of − 160‰. Moreover, the δ^15^N values of the nitrogen gases are quite negative in the range of − 0.1 to − 0.4‰.

**Figure 13:**
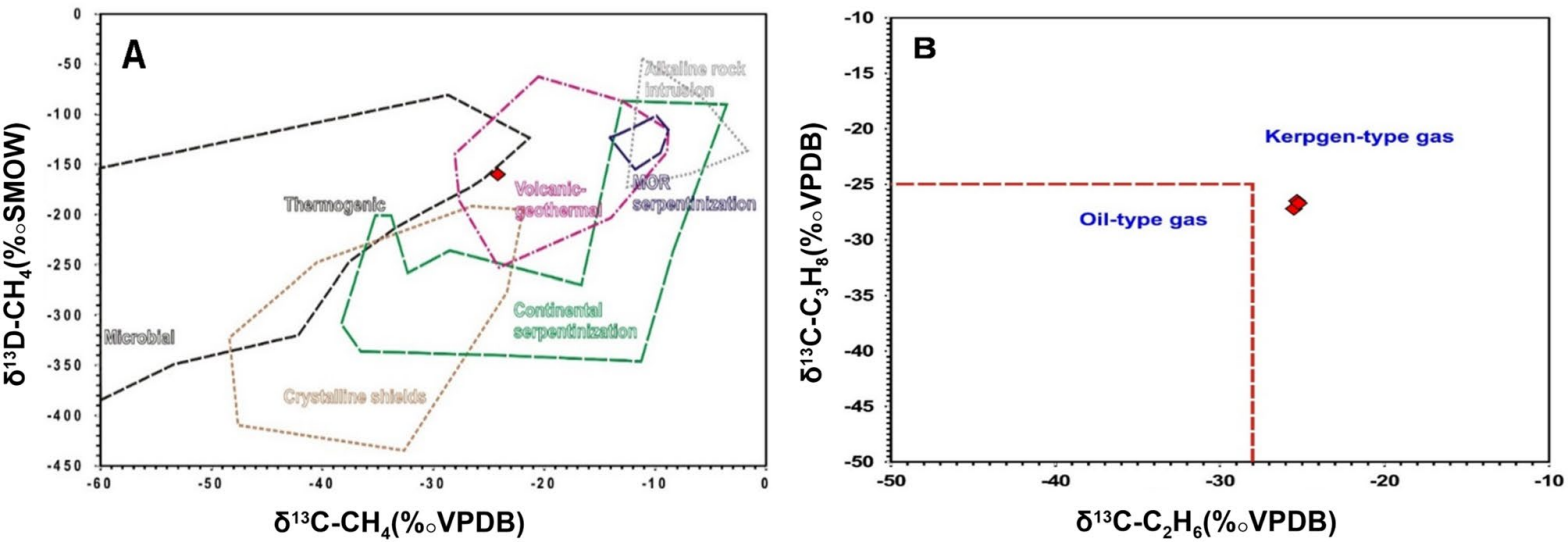
(**A**) The δD_CH4_ and δ^13^C_CH4_ gas origin diagram of ^10^, illustrating the mixed thermogenic volcanic–geothermal source of CH_4_ in the investigated gas seeps; (**B**) δ^13^C_C2H6_ versus δ^13^C_C3H8_ cross-plot, showing that the C2 + phases in the gas seeps were formed via thermogenic cracking of the kerogen.

The isotopic composition of the different gas phases often points to their origin and mechanism of generation (^6,10,48–52^and references therein). The measured δ^13^C_CO2_ values point to the organic origin of the CO_2_ gases due to the thermal decomposition of organic-rich sediments (Fig. 12A). Light δ^13^C_CO2_ values < −13‰ reveal minimal or no contribution from either volcanic or magmatic CO_2_, and light negative values preclude any input from the thermal decomposition of carbonate-rich sediments^5,6^. Similarly, the molecular versus isotopic composition of the hydrocarbon gas phases typifies a pure thermogenic origin of the hydrocarbon gases (Fig. 12B). This is further confirmed by the relatively wet composition of the hydrocarbon gases (C_1_/C_2_ + C_3_ < 100), as well as the elevated concentration of the C_6+_ hydrocarbons. The relatively heavy δ^13^C_CH4_ values coupled with low molecular ratios support hydrocarbon gas formation via thermal cracking of sediments enriched in type II kerogen capable of generating wet-type gases ^53^. Furthermore, the δ^13^C_CH4_ and δD_CH4_ plot correlates with gases generated by geothermal thermogenic processes (Fig. 13A). Additionally, the isotopic values of ethane and propane that do not correlate with those derived from oil cracking (oil-type gas) support their derivation from thermal cracking of the kerogen (Fig. 13B).

## Integrated results and discussion

Geomagnetic measurements indicated the existence of a closed anomaly with high values near the smoke exit area (SEA), shown as a yellow square in Fig. 3, that quickly declines to the northeast, which is linked to the presence of a fault. This result indicates that this fault's linear anomaly runs northwest‒southeast and connects with another primary fault (running northeast‒southwest) in the smoke exit area. The results of the VLF, as well as the results of the TEM, revealed that the area of smoke and high temperatures is characterized by a decrease in electrical resistivity compared to the rest of the areas, indicating that high temperature has an effect on electrical conductivity, causing it to increase and, as a result, reduce the electrical resistivity in the area of smoke and high temperatures. This process relies on the fact that the electric resistivity of aqueous solutions in the interstitial spaces in the rocks decreases with increasing temperature. This is because a decrease in the viscosity of water increases the mobility of ions^54^.

Using geophysical data, it was discovered that the study area is exposed to two main faults and that the smoke exit area is located at the intersection of these two faults. It was also discovered that the study area contains numerous cracks and fissures, which could have been caused by high temperatures. These faults and fractures act as routes and outlets for the rising of groundwater from aquifers; the groundwater that has risen aids in the reaction processes occurring in the smoke-bearing region inside the fault zone.

The temperature records obtained from the two wells adjacent to the research region exhibit typical temperature gradients that align with the range observed and computed in other studies conducted in the Western Desert^22,55,56^. Since these wells draw water from the shallow aquifer at a depth of approximately 300 m, they indicate that the groundwater is unaffected by the high temperature caused by the phenomenon under study and that this phenomenon occurs close to the Earth's surface.

**Figure 14:**
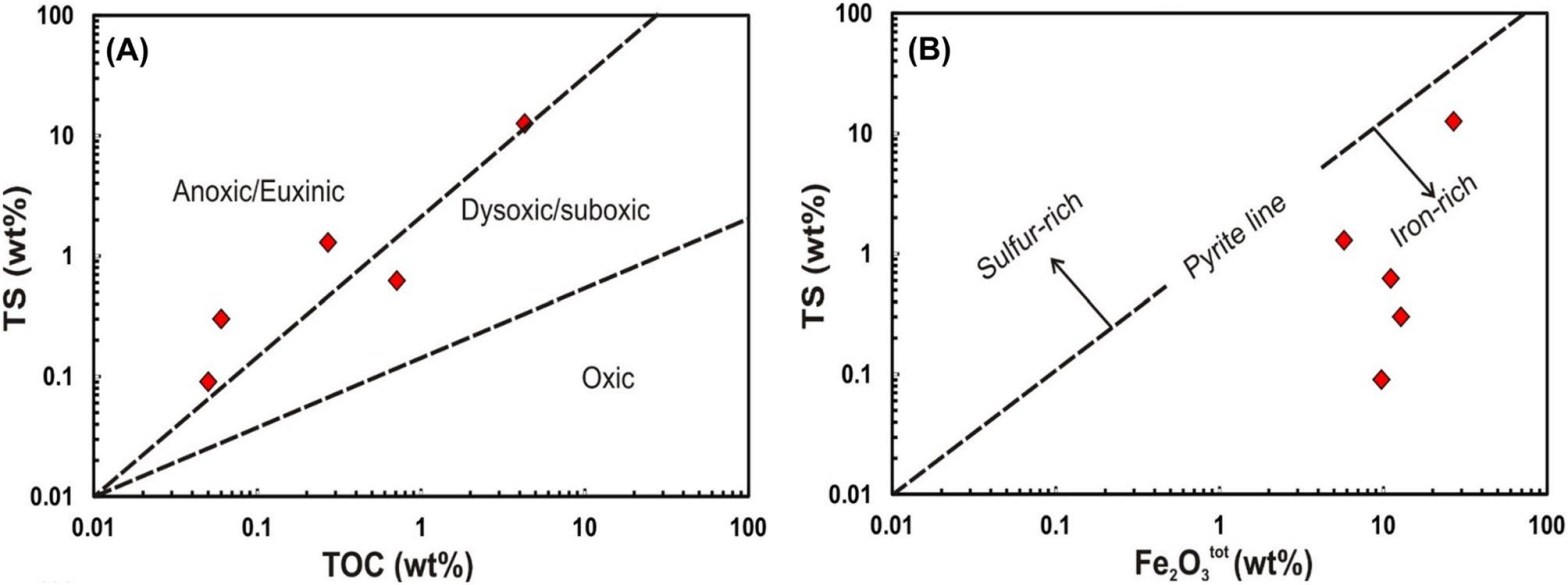
Total sulfur (TS) versus TOC (**A**) and total iron as Fe_2_O_3_ (**B**) for the studied Dakhla shale samples illustrate their iron-rich composition and deposition in an anoxic–suboxic depositional environment.

In terms of geochemistry, the studied gas seeps have been generated via near-surface reactions coupled with thermal degradation of materials rich in type II and II/III kerogen in Mut shales. Petrological data reveal abundant cuticles and amorphous organic matter in the fresh samples, typifying their ability to generate wet gases upon thermal maturation^57–59^. Additionally, the TOC and TS contents reveal that the studied organic-rich shales were deposited under anoxic bottom water, resulting in good preservation of the organic matter. (Fig. 14A). Sulfur was preferentially bound to the organic matter rather than reactive iron (Fig. 14B), resulting in the generation of sulfur-rich kerogen (type II S), typifying the assemblage of organic material in aerobic conditions in the presence of sulfate-reducing bacteria^60,61^.

**Figure 15:**
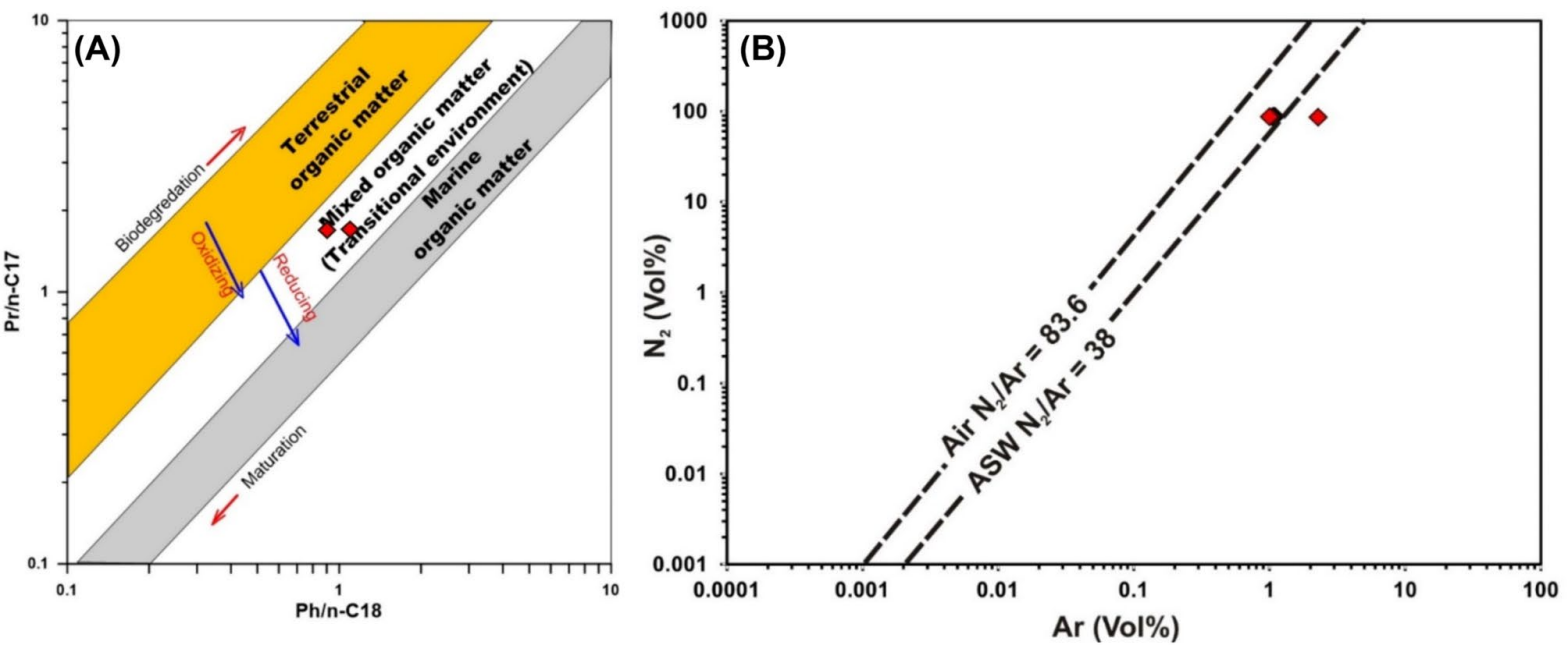
(**A**) Isoprenoid/n-alkane diagram of Shanmugam (1985)^64^ confirming the reducing depositional environment of the Dakhla shale bitumen extracts. (**B**) N_2_-Ar cross-plot highlighting their origin in the studied gas seeps. The N2 and Ar concentrations suggest their atmospheric origin.

The composition of bitumen extracts confirms the deposition of organic matter in a reducing environment (Fig. 15A). High-temperature catagenetic degradation of sulfur-rich kerogen would generate mainly CO_2_ and hydrocarbon gases in the range of C_1_–C_5_ coupled with the release of sulfur-rich compounds ^62,63^. Indeed, the chemistry of the studied gases and the precipitation of sulfur in the vicinity of the gas seeps are consistent with the hypothesis of gas generation via catagenetic cracking of sulfur-rich kerogen in the Mut shales.

**Figure 16:**
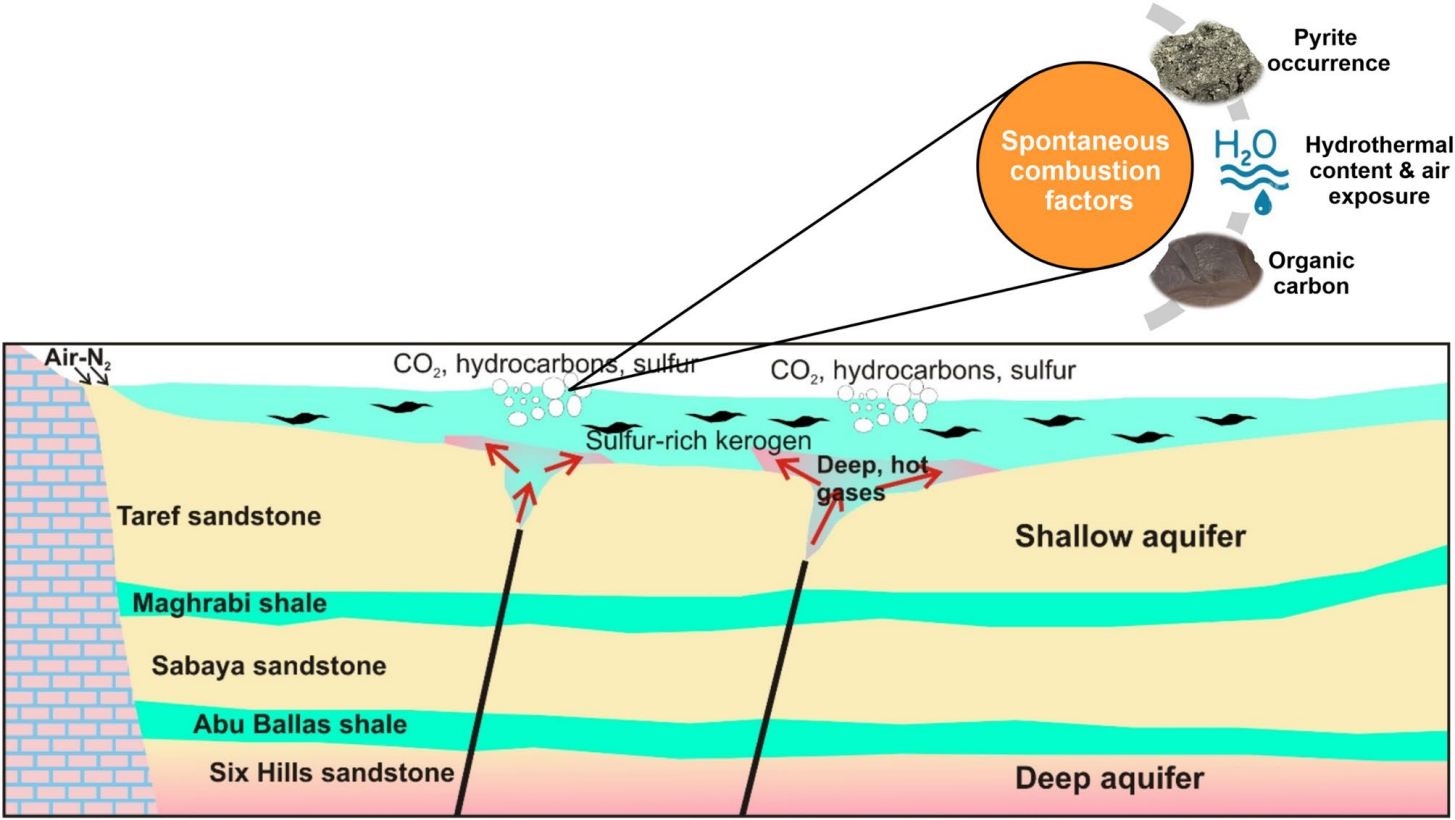
Illustrative diagram (not to scale) showing the formation mechanism of the studied gas seeps through a surficial interaction between the organic-rich shales and hot fluids. The interpreted deep-seated faults act as conduits for hot fluids from the deep sandstone aquifers to the surface.

Hot meteoric water circulating in the sandstone aquifers beneath the Mut shales likely promotes the catagenetic degradation of the kerogen. This is confirmed by the nitrogen versus argon plot (Fig. 15B), where the nitrogen and argon contents in the gas seeps are comparable to those of air and air-saturated water. Moreover, the δ^15^N values in the gases in a range close to atmospheric values (δ^15^N = 0) confirm the atmospheric origin of N_2_ gases, which were likely mixed with meteoric water in the sandstone aquifers of the Taref, Sabaya and Six Hills Formations. Therefore, we hypothesize that the deep-seated faults act as conduits for hot water and gases circulating in the sandstone aquifers into the overlying Mut shales, resulting in thermal degradation of the sulfur-rich kerogen and generation of the studied gas seeps (Fig. 16). Increasing water content and moisture causes a decrease in the solidity of rocks, which in turn causes variations in temperature through oxidation processes. The water content and inherent moisture content may have an impact on the likelihood of spontaneous combustion^65^.

The research findings demonstrated that a variety of factors work together to generate spontaneous combustion; none of these factors can cause continuous burning, such as that in the study region (Fig. 16). Initially, pyrite is oxidized, which is an exothermic process that creates heat, increasing the reaction rate, and pyrite oxidation results in the precipitation of sulfur and iron. The organic carbon inside the shale then functions as a fuel source for combustion, allowing long-term activity to occur once the requisite ignition temperature is attained. Organic carbon concentrations must be adequate to support combustion, and this research claims that TOC% levels greater than 4% are sufficient to sustain combustion for an extended period of time. When temperatures exceed 100 °C, the moisture content of the oil shale evaporates, causing the shale to expand, become brittle, and crack, increasing the surface area of combustion. A source of oxygen is necessary for combustion to proceed, which may be supplied by air arriving at the shale or by movement of convection water via fissures and cracks. As a result, oxygen flow is a critical aspect in pyrite oxidation and must be well adapted to the initiation and maintenance of combustion.

## Conclusion and recommendation

A strange phenomenon appeared in the village of Al-Hindaw in Dakhla, New Valley Governorate, which was reported by various media. It was the emission of gases and thick smoke rising out of the Earth, which surprised and alarmed everyone in the area. The research region was subjected to field measurements using a variety of geophysical instruments. In addition, geochemical analyses, organic petrography and organic geochemical analysis, as well as gas sampling and analyses, were conducted. Integration of these studies suggested that spontaneous combustion, high temperatures, and gas escape are generated by a combination of factors. Pyrite oxidation by exposure to air or groundwater and organic carbon combustion are two main reasons for continuous spontaneous combustion.

The release of gases and high temperatures in the village of Hindaw is caused by chemical reactions and not by volcanic or seismic activity; thus, there is no reason to be concerned about the stability of the Earth in the research area. The investigation demonstrated the presence of pyrite in the samples taken from the study region in a significant proportion (approximately 20%). Pyrite is an industrial mineral that is used in several industries. To evaluate the economic viability of extracting this mineral, we suggest performing integrated research in the Al-Hindaw region and its vicinity.

Residents of the village of Hindaw and visitors watching this phenomenon must be warned to follow the prohibition and to avoid breathing the produced gases, the majority of which are hydrogen sulfide gas, since inhaling significant quantities of this gas has a detrimental impact on the respiratory and neurological systems. It is advisable to backfill the gas departure area to minimize the escape of gas until optimal use is found (Supplementary information 1, 2 and 3).

### Supplementary Information

Supplementary Movie 1.

Supplementary Movie 2.

Supplementary Legends.

## Data Availability

The data that support the outcomes of this work are accessible upon reasonable request from the corresponding author.
