# Supplementary material for: Origin of mysterious geothermal gas emissions in the middle of the Western Desert, stable shelf area, Dakhla Oasis, Egypt
Source: Sci Rep. 2023 Sep 30;13:16466. doi: 10.1038/s41598-023-43492-1 (PMC10542370; doi:10.1038/s41598-023-43492-1)
Supplement: Supplementary file 3 — Supplementary Legends. [file 41598_2023_43492_MOESM3_ESM.docx]

Video 1. A clip depicting the massive release of smoke and fumes from the earth. It was necessary to collect a sample of these gases so that their composition could be analyzed and identified.

Video 2. A video clip depicting the emergence of fire and flames while moving soil with a bulldozer operated by the Dakhla City Council at the request of the work team to take a sample of the burnt rocks. The video clip was captured by one of the workers, and the guy who continuously appears in the video and speaks is a resident of the area who was the first to discover and post this phenomenon on his Facebook page. The teamwork was there at the event but was not captured on camera.
